# Thioredoxin Reductase Is Involved in Development and Pathogenicity in *Fusarium graminearum*

**DOI:** 10.3389/fmicb.2019.00393

**Published:** 2019-03-07

**Authors:** Xinyue Fan, Fang He, Mingyu Ding, Chao Geng, Lei Chen, Shenshen Zou, Yuancun Liang, Jinfeng Yu, Hansong Dong

**Affiliations:** Key Laboratory of Agricultural Microbiology, College of Plant Protection, Shandong Agricultural University, Tai’an, China

**Keywords:** *Fusarium graminearum*, thioredoxin reductase, development, pathogenicity, apoptosis

## Abstract

*Fusarium graminearum* is one of the causal agents of Fusarium head blight and produces the trichothecene mycotoxin, deoxynivalenol (DON). Thioredoxin reductases (TRRs) play critical roles in the recycling of oxidized thioredoxin. However, their functions are not well known in plant pathogenic fungi. In this study, we characterized a TRR orthologue FgTRR in *F. graminearum*. The FgTRR-GFP fusion protein localized to the cytoplasm. *FgTRR* gene deletion demonstrated that FgTRR is involved in hyphal growth, conidiation, sexual reproduction, DON production, and virulence. The Δ*TRR* mutants also exhibited a defect in pigmentation, the expression level of aurofusarin biosynthesis-related genes was significantly decreased in the *FgTRR* mutant. Furthermore, the Δ*TRR* mutants were more sensitive to oxidative stress and aggravated apoptosis-like cell death compared with the wild type strain. Taken together, these results indicate that *FgTRR* is important in development and pathogenicity in *F. graminearum*.

## Introduction

Fusarium head blight (FHB), caused mainly by *Fusarium graminearum*, is an importantly destructive disease of wheat and barley worldwide (Goswami and Kistler, 2004). The disease not only reduces wheat yield, but also endangers the health of humans and animals because of consumption of mycotoxin-contaminated grain (McMullen et al., 1997; Goswami and Kistler, 2004, 2005; Desjardins and Proctor, 2007). Deoxynivalenol (DON), one of trichothecene mycotoxins produced by *F. graminearum*, is recognized as an important virulence factor during plant infection (Desjardins et al., 1993, 1996; Proctor et al., 1995) and is a potent protein synthesis inhibitor (Pestka and Smolinski, 2005; Arunachalam and Doohan, 2013). The *TRI* gene cluster, including *TRI5, TRI6, TRI10*, and other *TRI* genes, are involved in DON synthesis in *F. graminearum* (Proctor et al., 1995; Brown et al., 2004; Alexander et al., 2009).

The thioredoxin system, comprised of thioredoxin reductase (TRR), thioredoxin (TRX), and NADPH, is involved in regulating methionine biosynthesis, cell growth, gene transcription, and apoptosis (Lindner et al., 2002; Koc et al., 2006; Bobba et al., 2014). TRR is a member of the pyridine nucleotide-disulfide oxidoreductase family and usually includes FAD-and NADPH-binding domains and an active site containing a redox-active disulfide (Williams, 1995; Mustacich and Powis, 2000; Arnér, 2009). Hallmarks of apoptosis include externalization of phosphatidylserine(PS), DNA fragmentation, and intracellular reactive oxygen species (ROS) accumulation. In microbial eukaryotes, ROS, including superoxide anion (O^2-^), hydrogen peroxide (H_2_O_2_), and hydroxyl radical (OH^-^), are involved in immunization, cell proliferation, signal transduction, and other biochemical reactions (Aguirre et al., 2005; Tudzynski et al., 2012). The cellular redox regulation is achieved mainly through the thioredoxin system (Powis et al., 2000; Bobba et al., 2014).

Yeast contains two genes encoding TRRs (cytoplasmic TRR1 and mitochondrial TRR2), and deletion of *TRR1* increased sensitivity to oxidative and reductive stress, high temperature, and auxotrophic requirement for methionine (Pearson and Merril, 1998; Carmel-Harel et al., 2001; Trotter and Grant, 2002). In addition, *TRR1* affects the transcription of cell cycle-regulated genes at the G1/S boundary and the activity of the p53 tumor suppresser gene in yeast (Machado et al., 1997; Pearson and Merril, 1998). In *Acremonium chrysogenum*, TrxR1 (TRR1) is required for the normal growth and related cephalosporin C production in methionine-supplemented medium (Liu et al., 2012). Characterization of the role of TRR in phytopathogenic fungi, *Magnaporthe oryzae*, and *Botrytis cinerea*, showed that TRR is involved in growth and virulence (Fernandez and Wilson, 2014; Viefhues et al., 2014).

In this study, we hypothesized that *FgTRR* may play important roles in development and virulence in *F. graminearum*. Therefore, we characterized the role of *FgTRR* in *F. graminearum*. Deletion of *FgTRR* led to defects in hyphal growth, asexual and sexual reproduction, pathogenicity, and DON production, indicating that FgTRR is involved in regulating development and virulence in *F. graminearum*.

## Materials and Methods

### Strains and Culture Conditions

*Fusarium graminearum* strain PH-1 was used as the wild-type (WT) strain in this study. The WT and its derivative mutants were routinely cultured on potato dextrose agar (PDA) and complete medium (CM) at 25°C for mycelial growth tests. Liquid carboxymethyl cellulose (CMC) medium was used to analyze induction of asexual reproduction (Hou et al., 2002). For determining sensitivity to various stresses, mycelial growth was assayed on CM plates supplemented with 0.7 M NaCl, 0.7 M KCl, 0.2 g/L Congo Red (CR), 1 M sorbitol, 0.05% SDS, 5 mM H_2_O_2_, and 15 μM menadion. Fungal mycelia were harvested from potato dextrose broth (PDB) and used for extraction of genomic DNA and RNA. *Escherichia coli* DH5α was used for routine transformations and subsequently cultured in Luria-Bertani broth at 37°C.

### Generation of *FgTRR* Deletion Mutants

The split-marker approach (Catlett et al., 2003) was used to generate the *FgTRR* deletion mutants. Briefly, the 1422-bp upstream and 1061-bp downstream flanking sequences were amplified with primer pairs TRR-A1/TRR-A2 and TRR-B1/TRR-B2 (Supplementary Table S1), respectively. The amplicon containing hygromycin phosphotransferase (hph) was amplified from the pCB1003 with primer pairs HYG-F/HY-R and YG-F/HYG-R (Supplementary Table S1). *FgTRR* gene-replacement constructs were generated by overlapping PCR. Then, the resulting constructs were directly transformed into protoplasts of the WT strain with PEG-mediated transformation, and transformants were selected on TB3 medium with the final concentration of 200 μg/mL of Hygromycin B, and further identified by PCR and Southern blot analysis (Supplementary Table S1 and Supplementary Figure S2).

### Generation of Δ*TRR* Complementation and Subcellular Localization

The yeast gap repair approach (Bruno et al., 2004) was used to generate the Δ*TRR* complementation (Δ*TRR*-C) strain. Briefly, a 3.3-kb DNA fragment from the *FgTRR* gene carrying its native promoter was amplified with primer TRR-CF/TRR-CR (Supplementary Table S1) and co-transformed with *Xho*I-digested pYF11 vector harboring GFP into *S. cerevisiae* XK1-25. The resulting construct was directly transformed into protoplasts of the *FgTRR* deletion mutant. Transformants were selected with 200 μg/mL G418 and identified by PCR with the primer pair TRR-CF/TRR-CR (Supplementary Table S1 and Supplementary Figure S2). GFP signals in hyphae were visualized with a laser confocal microscope (LSM880NLO, ZEISS).

### Asexual and Sexual Reproduction Assays

Conidiation was examined in CMC medium after 5 days of incubation at 25°C (Hou et al., 2002). The number of conidia were counted for each strain using a haemocytometer. For sexual reproduction assays, aerial hyphae were gently removed on carrot agar (CA) plates after adding with 0.1% Tween 20 solution (Bowden and Leslie, 1999; Cavinder et al., 2012). Perithecia and ascospores were examined after 2–3 weeks of incubation at 25°C. The experiments were repeated three times with three replicates each time.

### Plant Infection and DON Production Assays

Virulence assays were performed on wheat cv. Jimai 22 during flowering. A 10-μL suspension of conidia (4.0 × 10^5^ conidia/mL) was injected into a floret in the central section of wheat ear (Gale et al., 2002; Han et al., 2004). Disease index was assayed 14 days post-inoculation. For infection of corn silks, mycelial plugs (5 mm in diameter) taken from PDA plates were inoculated on a group of silks and kept for 5 days at 25°C as described previously (Seong et al., 2005). For assays of DON production, 5 g of rice grains in per flask were autoclaved and inoculated with three mycelial plugs per flask. After 20 days of incubation at 25°C, DON concentrations were determined by a liquid chromatography-mass spectrometer/mass spectrometer (HPLC–MS/MS) system (AB Sciex, 5500) (Soleimany et al., 2012; Zheng et al., 2012). Ergosterol levels were used to normalize DON production per fungal mass. The experiments were repeated three times.

### DNA Isolation and Southern Blot

Genomic DNA was extracted from mycelia as described previously (Leslie and Summerell, 2006) and digested with *Nde*I. Southern blot analysis was performed with the DIG-High Prime DNA Labeling and Detection Starter kit I according to the manufacturer’s protocol (Roche Diagnostics, Mannheim, Germany). The probe was amplified by PCR using the primer pair tTRR-F/tTRR-R (Supplementary Table S1).

### RNA Extraction and qRT-PCR Analysis

Total RNAs were extracted from mycelia grown in PDB or DON-inducing medium (Gardiner et al., 2009) for 3 days using the RNAiso Plus reagent (TaKaRa, TaKaRa Biotechnology Co., Dalian, China). cDNA was synthesized by PrimeScript^TM^ RT reagent as instructed by the manufacturer (TaKaRa Biotechnology). The expression level of genes related to aurofusarin and DON synthesis were detected by qRT-PCR with the primers listed in Supplementary Table S1. Relative quantification of each transcript was calculated by the 2^-ΔΔCT^ method (Livak and Schmittgen, 2001).

### Analysis of Phosphatidylserine Externalization and Intracellular ROS Accumulation

Protoplasts were stained with the Annexin V-FITC Apop kit (Invitrogen, United States) to analyze PS externalization (Madeo et al., 1997). Briefly, protoplasts of WT and Δ*TRR*#*1* were resuspended in 1.2 M KCl and treated with 5 mM H_2_O_2_ for 1 h. After washing protoplasts with 1.2 M KCl and resuspending in 200 μL binding buffer (1×), a 5-μL Annexin V-FITC was added into 195 μL of the protoplast suspension and the mixture was incubated for 10 min at room temperature. Then, after washing protoplasts with 200 μL of binding buffer (1×) and resuspending again in 190 μL of binding buffer (1×), 10-μL of propidium iodide (PI) (20 μg/mL) was added into the protoplast suspension and incubated for 15 min in the dark. Protoplasts were resuspended in 1.2 M KCl and analyzed by fluorescence microscopy. The experiments were repeated three times with three replicates each time.

Intracellular ROS accumulation was measured with a fluorescent probe, dihydrorhodamine (DHR) 123 (Sigma–Aldrich, United States) as previously described (Royall and Ischiropoulos, 1993). The mycelia cultured in liquid YEPD for 18 h were collected and stained with 5 μM DHR123 for 30 min, rinsed, and re-suspended in PBS. Fluorescence intensity was visualized by fluorescence microscopy.

### Statistical Analysis

Data are presented as means ± standard deviations from three independent experiments. Different letters and asterisks indicate a significant difference (*P* < 0.05) by Duncan’s multiple range test or Student’s *t*-tests.

## Results

### Identification and Subcellular Localization of FgTRR in *F. graminearum*

**FIGURE 1:**
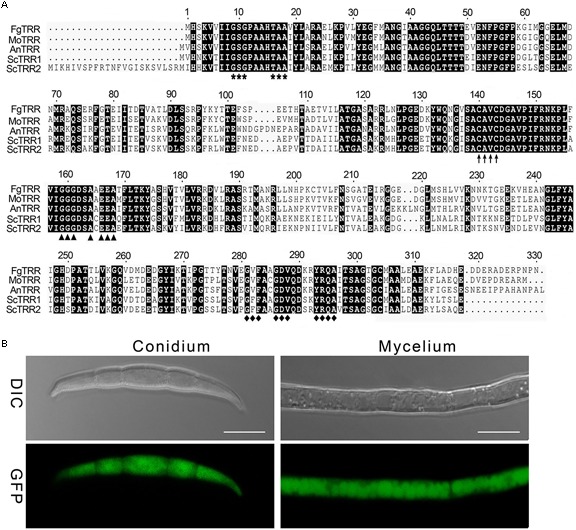
Sequence alignment and phylogenetic analysis of thioredoxin reductases (TRRs) in fungi. **(A)** Sequence alignment of FgTRR with other fungal TRRs. Fg, *Fusarium graminearum*; Mo, *Magnaporthe oryzae*; An, *Aspergillus nidulans*; Sc, *Saccharomyces cerevisiae*. Bold asterisks, arrows, triangles, and rhombuses denote a FAD-binding domain I (GXGXX), a redox-active cysteine pair (CAVC), a NADPH-binding domain (GGGXXA), and a FAD-binding domain II (GXFAXGDV), respectively. **(B)** Subcellular localization of the FgTRR-GFP fusion protein in *F. graminearum.* GFP signal observation in hyphae and conidia was determined using a laser confocal microscope. Bar = 10 μm.

One putative TRR (FGSG_00871, designated as FgTRR) in *F. graminearum* was retrieved by BLAST search of the NCBI with the *M. oryzae* TRR1 (MGG_01284) as a query. The *FgTRR* gene is predicted to encode a 330-amino acid protein showing 46% identity to *S. cerevisiae* TRR1 and 79% to *M. oryzae* TRR1. The domain analysis showed that FgTRR was predicted to harbor all domains typical for the TRR family, including a FAD-binding domain I (GXGXX, where X is any amino acid), a FAD-binding domain II (GXFAXGDV), and a NADPH-binding domain (GGGXXA). Additionally, it contains a redox-active cysteine pair (CAVC) (Figure 1A). Phylogenetic analysis by the neighbor-joining method revealed that TRR is highly conserved among three *Fusarium* species (Supplementary Figure S1).

To explore the subcellular localization of FgTRR, we generated the FgTRR-GFP fusion protein by using its native promoter. Transformants were validated by selection on G418-containing medium and PCR analysis (Supplementary Figure S2). Bright green fluorescence was observed and distributed uniformly throughout hyphae and conidia (Figure 1B). Therefore, we assumed that FgTRR localized to the cytoplasm.

### FgTRR Is Responsible for Hyphal Growth, Conidiation, and Conidial Germination

To study the function of the *FgTRR* gene in *F. graminearum*, we generated the *FgTRR* deletion mutants by replacing the targeted coding region with the *hph* gene. After hygromycin screening, seven independent putative *FgTRR* deletion mutants were verified by PCR and confirmed by Southern blot hybridization (Supplementary Table S1 and Supplementary Figure S2). For complementation, we performed *FgTRR* complementation (Δ*TRR-*C) by reintroducing the entire *FgTRR* gene under the control of its native promoter into the Δ*TRR*#*1* mutant, one of *FgTRR* gene deletion mutants.

**FIGURE 2:**
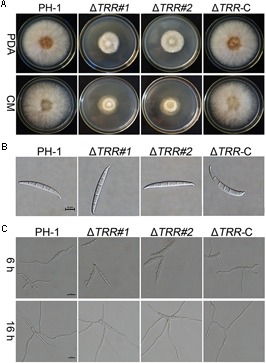
Hyphal growth, conidial morphology, and germination of the *FgTRR* deletion mutants. **(A)** Colonies of the wild type (PH-1), *FgTRR* deletion mutants, and Δ*TRR-*C strains were cultured on potato dextrose agar (PDA) and complete medium (CM) agar plates. Photos were recorded after growth for 3 days at 25°C. **(B)** Conidial morphology of the Δ*TRR* mutants. Conidia were examined by differential interference contrast microscopy. Bar = 10 μm. **(C)** Conidia of the Δ*TRR* mutants were incubated in liquid YEPD at 25°C and examined for germination. Bar = 20 μm.

In comparison with the WT and Δ*TRR-*C strain, the Δ*TRR* mutants displayed reduced hyphal growth rates on PDA and CM plates. The mutants were reduced by approximately 56% on PDA and 60% on CM, respectively (Table 1 and Figure 2A). The *FgTRR* mutants produced fewer conidia but with normal morphology (Table 1 and Figure 2B). We then investigated the germination of conidia. After 6 h post-incubation (hpi) in YEPD medium, approximately 6% of the Δ*TRR* conidia germinated but the germination rate of the WT was over 97% under the same conditions. In addition, compared with the WT, the length of germ tube was obviously decreased in the Δ*TRR* mutants (Figure 2C). After 16 hpi, the germination rate of the Δ*TRR* mutants showed a slight decrease compared with the WT. Taken together, FgTRR is involved in vegetative growth, conidiation, and conidial germination.

**Table 1:** Hyphal growth, conidiation, and DON production of the *FgTRR* deletion mutants^∗^.

Strain	Colony diameter (cm)^α^		Conidiation (×10^6^ spores/ml)^β^	Conidial germination (%)^γ^		DON/Erg^δ^
	PDA	CM		6 h	16 h	
WT	7.42 ± 0.05^a^	7.40 ± 0.05^a^	3.63 ± 0.05^a^	97.97 ± 0.02^a^	99.41 ± 0.24^a^	0.99 ± 0.02^a^
Δ*TRR#1*	3.12 ± 0.10^b^	3.03 ± 0.05^b^	2.85 ± 0.12^b^	5.05 ± 0.35^b^	97.13 ± 0.60^b^	0.03 ± 0.003^b^
Δ*TRR#2*	3.30 ± 0.09^b^	3.03 ± 0.11^b^	2.97 ± 0.08^b^	4.68 ± 0.26^b^	97.71 ± 0.19^b^	0.04 ± 0.004^b^
Δ*TRR-*C	7.27 ± 0.13^a^	7.27 ± 0.07^a^	3.59 ± 0.09^a^	97.09 ± 0.25^a^	99.53 ± 0.27^a^	1.07 ± 0.07^a^

*^∗^Means and standard deviations were calculated from three independent experiments. Different superscript letters indicate a significant difference (*P* < 0.05) by Duncan’s multiple range test. ^α^Colonies were cultured on PDA or CM plates for 3 days at 25°C. ^β^Conidiation in 100 ml of carboxymethylcellulose (CMC) cultures was examined after incubation for 5 days. ^γ^Conidial germination was assayed after 6 and 16 h of incubation at 25°C. ^δ^DON and ergosterol (Erg) production was analyzed by HPLC-MS/MS. Rice samples inoculated with mycelial plugs were cultured for 20 days at 25°C.*

### FgTRR Is Involved in Ascus and Ascospore Development

**FIGURE 3:**
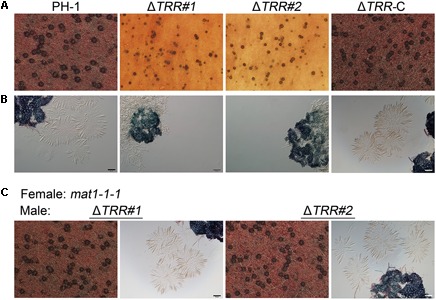
Assays for sexual reproduction in the *FgTRR* deletion mutants. **(A)** Perithecia produced by PH-1, the Δ*TRR* mutants, and Δ*TRR-*C at 14 days post fertilization. **(B)** Asci and ascospores observed under a microscope after squeezing perithecia. **(C)** Asci and ascospores from the Δ*TRR* mutants (male) × the *mat1-1-1* strain (female). Bar = 50 μm.

In FHB development, ascospore formation is crucial for plant infection. Therefore, we further investigated the ability of the Δ*TRR* mutants in sexual reproduction. We found that the WT and Δ*TRR-*C strains produced many perithecia and normal ascospores on CA plates. Although perithecia of the Δ*TRR* mutants were observed, there were no asci and ascospores released in the Δ*TRR* mutants even after 30 days of induction (Figure 3A,B), indicating deletion of *FgTRR* blocks ascus development but has no effect on the formation of protoperithecia. In order to further determine whether the Δ*TRR* mutants are male fertile, the Δ*TRR* mutants were crossed as the male with the *mat1-1-1* strain (Zheng et al., 2013). As shown in Figure 3C, asci and ascospores were normally produced in the Δ*TRR* mutants after mating with the *mat1-1-1* strain, indicating that *FgTRR* mutants are male fertile in *F. graminearum*.

### *FgTRR* Is Essential for Pathogenicity and DON Production

**FIGURE 4:**
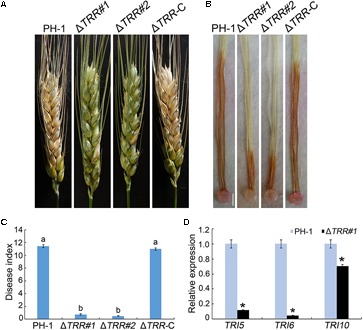
Pathogenicity and *TRI* expression assays of the *FgTRR* deletion mutants. **(A)** Flowering wheat heads were inoculated with conidial suspensions and examined 14 days post-inoculation (dpi). **(B)** Corn silks were inoculated with mycelial plugs and examined 5 dpi. Bar = 5 mm. **(C)** Disease index was examined from the number of symptomatic spikelets per wheat head 14 dpi. Different letters indicate statistically significant difference (*P* < 0.05) by Duncan’s multiple range test. **(D)** Relative expression levels of *TRI5, TRI6*, and *TRI10* in PH-1 and the Δ*TRR*#*1* mutant. The *GAPDH* gene was used as an internal control. Asterisks represent statistically significant difference (*P* < 0.05) by Student’s *t*-tests.

Pathogenicity of the mutants was assayed by inoculation of wheat heads with 4.0 × 10^5^ conidia/mL. As shown in Figure 4A, at 14 days post-inoculation (dpi), typical lesions spread from the inoculation site to other parts in wheat heads inoculated with the WT and Δ*TRR-*C strains. However, the Δ*TRR* mutants failed to develop, and the disease lesion was restricted to the inoculation site. Pathogenicity of the Δ*TRR* mutants was decreased by approximately 94% compared with the WT (Figure 4C). Similarly, the Δ*TRR* mutants caused only limited brown lesions on corn silks at 5 dpi (Figure 4B). These results confirm that *FgTRR* is important for virulence in *F. graminearum*.

In view of pathogenicity defects, we assayed DON production in the WT and Δ*TRR* mutants on autoclaved rice grains in flasks. As shown in Table 1, DON/ergosterol ratio of the Δ*TRR* mutants was significantly reduced compared to the WT. Furthermore, the expression of *TRI* (*TRI5, TRI6*, and *TRI10*) genes, which are essential for the regulation of DON biosynthesis, were assayed by qRT-PCR. The expression levels of *TRI5, TRI6*, and *TRI10* in the Δ*TRR#1* mutant were reduced by approximately 9-, 21-, and 1.5-fold relative to the WT, respectively (Figure 4D).

### Deletion of *FgTRR* Results in Defective Pigmentation

**FIGURE 5:**
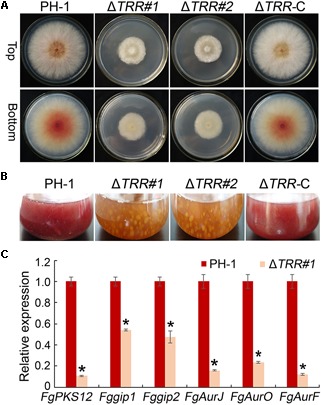
Defective pigmentation of the *FgTRR* deletion mutants. **(A)** Colony morphology on PDA plates 3 days post-incubation (dpi) at 25°C. **(B)** Liquid culture in flasks containing 50 mL of PDB medium 3 dpi at 25°C. **(C)** Relative expression levels of aurofusarin biosynthesis-related genes *FgAurJ* (*FGSG_02326*), *FgAurO* (*FGSG_02321*), *FgAurF* (*FGSG_02327*), *FgPKS12* (*FGSG_12040*), *Fggip1* (*FGSG_02328*), and *Fggip2* (*FGSG_02320*). The *GAPDH* gene was used as an internal control. Asterisks indicate statistically significant difference (*P* < 0.05) by Student’s *t*-tests.

When all strains were incubated on PDA plates for 3 days, the WT formed pink colonies, whereas the *FgTRR* mutants formed slightly pale yellowish colonies visible on the bottom of plates (Figure 5A). In PDB flasks, the Δ*TRR* mutants, similarly, exhibited the yellowish pigment (Figure 5B). These results showed that FgTRR is involved in the pigment biosynthesis in *F. graminearum*. To further confirm this conclusion, we performed qRT-PCR to determine the expression levels of *FgPKS12, Fggip1, Fggip2, FgAurJ, FgAurF*, and *FgAurO* that are related the biosynthesis of pigment aurofusarin (Kim et al., 2005, 2006; Frandsen et al., 2006). As shown in Figure 5C, the expression levels of six genes in the WT were significantly higher than those of the Δ*TRR#1* mutant.

### *FgTRR* Is Involved in Sensitivity to Various Stresses

**FIGURE 6:**
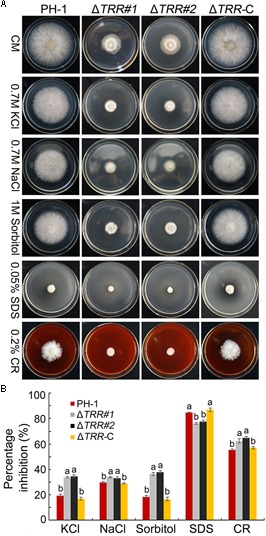
Sensitivity of the *FgTRR* deletion mutants to osmotic and cell wall-damaged stresses. **(A)** CM plates were supplemented with various stress agents at indicated concentrations. Photographs were taken after incubation for 3 days at 25°C. **(B)** Percentage inhibition was examined after incubation for 3 days. Means and standard deviations were calculated from three replicates. Different letters denote statistically significant differences (*P* < 0.05) by Duncan’s multiple range test.

In order to test the role of *FgTRR* in various stresses, we examined the sensitivity related to osmotic stress, oxidative stress, and cell wall stress. On CM plates containing sorbitol, the growth rate of the Δ*TRR* mutants was obviously inhibited compared to the WT. Similar trends in relative growth rates were observed on media supplemented with KCl or NaCl, indicating that the Δ*TRR* mutants are more sensitive to osmotic stress. The mutants exhibited sensitivity to the cell wall inhibitor CR and tolerance to SDS which causes cell membrane damage (Figure 6A,B). These results indicate that *FgTRR* is involved in stress response and cell wall integrity.

**FIGURE 7:**
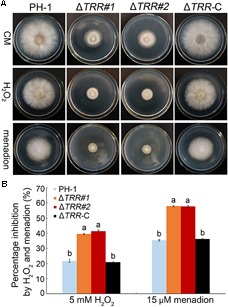
Sensitivity of the *FgTRR* deletion mutants to oxidative stress. **(A)** PH-1, Δ*TRR* mutants, and Δ*TRR-*C strains were cultured on CM plates supplemented with 5 mM H_2_O_2_ and 15 μM menadion. Photographs were taken after incubation for 3 days at 25°C. **(B)** Percentage inhibition was examined after incubation for 3 days. Means and standard deviations were calculated from three replicates. Different letters denote statistically significant differences (*P* < 0.05) by Duncan’s multiple range test.

The Δ*TRR* mutants were incubated on CM plates supplemented with H_2_O_2_ and menadion to confirm the sensitivity to oxidative stress. As shown in Figure 7A,B, the inhibition rate was significantly increased in mutants compared with the WT, indicating that *FgTRR* deletion mutants are more sensitive to oxidative stress.

### *FgTRR* Deletion Mutant Aggravates Apoptosis-Like Cell Death

**FIGURE 8:**
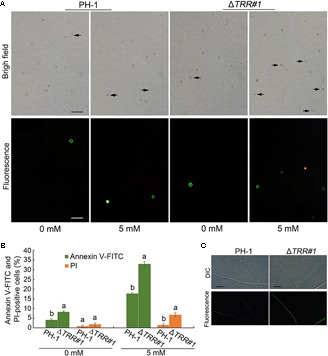
*FgTRR* is involved in apoptosis-like cell death in *F. graminearum*. **(A)** Protoplasts were treated with 5 mM H_2_O_2_ for 1 h and stained with Annexin V-FITC and propidium iodide (PI). Arrows indicate green and red cells showing Annexin V-FITC and PI-positive cells, respectively. Bar = 10 μm. **(B)** Percentage of Annexin V-FITC and PI-positive cells. At least 200 cells were counted in each repeat. Different letters denote statistically significant differences (*P* < 0.05) by Student’s *t*-tests. **(C)** The accumulation of intracellular ROS in hyphae of PH-1 and the Δ*TRR*#*1* mutant. Hyphae cultured at 25°C for 18 h in liquid YEPD were collected and stained with 5 μM DHR123. Bar = 20 μm.

To test the role of FgTRR in apoptosis-like cell death in *F. graminearum*, we first examined the extent of PS externalization in WT and Δ*TRR#1* by using Annexin V-FITC and PI staining. When treated with 5 mM H_2_O_2_ for 1 h, as shown in Figure 8A, protoplasts of the WT and the Δ*TRR#1* mutant were frequently stained green, indicating that these protoplasts showed PS externalization, a typical marker of early apoptosis-like cell death. The proportion of Annexin V-FITC-positive protoplasts was approximately 18 and 33% in the WT and the Δ*TRR#1* mutant treated with H_2_O_2_ for 1 h, respectively (Figure 8B). Similarly, in the absence of H_2_O_2_ treatment the proportion of Annexin V-FITC-positive protoplasts in the Δ*TRR#1* mutant was higher than that of the WT (Figure 8B). We next measured the accumulation of intracellular ROS in hyphae of the Δ*TRR#1* mutant. The results showed a stronger fluorescence in the Δ*TRR#1* mutant compared with the WT (Figure 8C). These data indicate that FgTRR is negatively involved in apoptosis-like cell death in *F. graminearum.*

## Discussion

The thioredoxin system consisting of TRR, TRX, and NADPH is ubiquitously present from yeast to filamentous fungi. In this study, we identified a TRR orthologue from *F. graminearum*. Sequence alignment revealed that FgTRR has features typical of TRRs from other fungi. The yeast *S. cerevisiae* contains two TRRs, TRR1 localizes in the cytoplasm and TRR2 in the mitochondrial matrix (Pedrajas et al., 1999; Carmel-Harel et al., 2001; Trotter and Grant, 2002), whereas only one TRR exists in some fungi (Fernandez and Wilson, 2014; Viefhues et al., 2014). Like yeast TRR1, FgTRR-GFP localizes to the cytoplasm, indicating that FgTRR is a cytosolic TRR.

The thioredoxin system is responsible for the redox regulation of DNA synthesis, development, gene transcription, and apoptosis (Lindner et al., 2002; Masutani et al., 2002; Koc et al., 2006). In this study, the *FgTRR* deletion mutants displayed a reduced hyphal growth rate, which is consistent with the *TRR1* in *B. cinerea* (Viefhues et al., 2014). *Cryptococcus neofoemans TRR1* is even essential for survival (Missall and Lodge, 2005). MoTrxs, a member of the thioredoxin system, play important roles in development, ROS signaling, and virulence in *M. oryzae* (Zhang S.J. et al., 2016). In contrast, *M. oryzae TRR1* mutants exhibited normal radial growth (Fernandez and Wilson, 2014). Consistent with TRR in *M. oryzae* (Fernandez and Wilson, 2014), conidiation of the *FgTRR* mutants was also significantly reduced after a 5-day incubation in liquid CMC medium, conidial germination rates of the *FgTRR* mutants were also reduced compared with the WT. Therefore, the *TRR* may have a conserved role in normal vegetative growth and conidiation. Interestingly, the Δ*TRR* mutants were defective in ascus and ascospore development but produced protoperithecia on CA plates. Like *TUB1* in *F. graminearum* (Chen et al., 2018), *FgTRR* is dispensable for initial stages of perithecium formation. Similarly, FgNoxR, a regulatory subunit of NADPH oxidases, is essential for perithecium formation in *F. graminearum* (Zhang C.K. et al., 2016). We propose that the thioredoxin system is required for sexual reproduction in *F. graminearum*.

Phytopathogenic species of *Fusarium* produce the red pigment aurofusarin, a secondary naphthoquinone metabolite (Medentsev and Akimenko, 1998; Frandsen et al., 2006). In this study, compared with the WT strain, the *FgTRR* mutants displayed reduced accumulation of red pigment, indicating that *FgTRR* deletion affects aurofusarin biosynthesis. The red coloration of CA plates was also reduced, which may be related to the downregulation of aurofusarin. Consistently, many genes including *Gip, PKS*, and *Aur*, which are responsible for aurofusarin production (Kim et al., 2005, 2006; Frandsen et al., 2006), were significantly decreased in the *FgTRR* mutant. The production of DON, an important virulence factor in *F. graminearum* (Desjardins et al., 1993, 1996; Proctor et al., 1995), was significantly decreased in the *FgTRR* mutants. Furthermore, qRT-PCR assays confirmed that *FgTRR* positively affected the expression of trichothecene biosynthesis genes *TRI5, TRI6*, and *TRI10.* TRR has been shown to be required for virulence in *B. cinerea* and *M. oryzae* (Fernandez and Wilson, 2014; Viefhues et al., 2014). We also found that deletion of *FgTRR* gene results in a defect in pathogenicity. Therefore, these results suggest that *FgTRR* is involved in secondary metabolism and pathogenicity.

Recently, it has been shown that deletion of *MoTRR* resulted in increased sensitivity to CR in *M. oryzae* (Fernandez and Wilson, 2014). In this study, we also found that the *FgTRR* mutants were more sensitive to CR, indicating that deletion of *FgTRR* decreases cell wall integrity in *F. graminearum*. The *FgTRR* mutants showed increased sensitivity to osmotic and oxidative stresses, like in other fungi (Carmel-Harel et al., 2001; Fernandez and Wilson, 2014; Viefhues et al., 2014). The pigments produced by fungi may provide protection against excessive oxidative stress (Rangel et al., 2015), we propose that, due to reduced accumulation of pigment, deletion of *FgTRR* results in the decreased ability to remove excessive H_2_O_2_. Taken together, FgTRR is important for response to various stresses.

Fungal apoptosis-like cell death is essential for normal development and maintenance of multicellular organisms, and fungi undergo apoptosis-like cell death in response to antifungal drugs or stresses (Shlezinger et al., 2012; Gonçalves et al., 2017). Apoptosis-like cell death in filamentous fungi has been identified as a crucial component in the infection of plants. Overexpression of the anti-apoptotic gene *BcBIR1* in *B. cinerea* confers enhanced pathogenicity and resistance to cell death (Shlezinger et al., 2011). Interestingly, deletion of *FgTRR* resulted in the accumulation of intracellular ROS, which ultimately aggravated apoptosis-like cell death. Therefore, these results indicate that *FgTRR* is responsible for apoptosis-like cell death in *F. graminearum*.

## Conclusion

In summary, our results indicate that the FgTRR protein plays a pleiotropic role in *F. graminearum*, including the regulation of vegetative growth, asexual and sexual reproduction, stress responses, pathogenicity, and DON synthesis. Furthermore, FgTRR is involved in apoptosis-like cell death in *F. graminearum*.

## Author Contributions

XF, FH, and MD conceived and designed the experiments. XF, FH, MD, CG, LC, and SZ performed the experiments. LC and SZ analyzed the data. XF, FH, and MD wrote the manuscript. YL, JY, and HD originated research leading up to this paper and provided guidance and review.

## Conflict of Interest Statement

The authors declare that the research was conducted in the absence of any commercial or financial relationships that could be construed as a potential conflict of interest.
