# Supplementary material for: Thioredoxin Reductase Is Involved in Development and Pathogenicity in Fusarium graminearum
Source: Front Microbiol. 2019 Mar 7;10:393. doi: 10.3389/fmicb.2019.00393 (PMC6416177; doi:10.3389/fmicb.2019.00393)
Supplement: Supplementary file 1 [file Data_Sheet_1.docx]

**Thioredoxin reductase is involved in development and pathogenicity in *Fusarium graminearum***

Xinyue Fan, Fang He, Mingyu Ding, Cao Geng, Lei Chen, Shenshen Zou, Yuancun Liang and Jinfeng Yu

Key Laboratory of Agricultural Microbiology, College of Plant Protection, Shandong Agricultural University, Tai’an 271018, China

**Correspondence**: E-mail: [liangyc@sdau.edu.cn](mailto:liangyc@sdau.edu.cn)

**
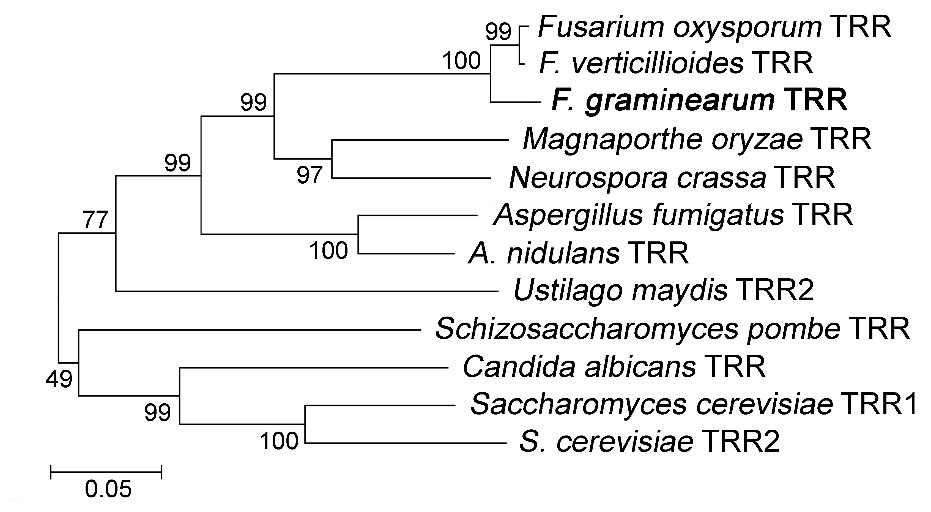
**

**FIGURE S1 |** Phylogenetic analysis of TRR from fungi using MEGA 6.0 software. Fungal species and protein accession numbers: *Fusarium graminearum* TRR (FGSG_00871), *F. oxysporum* TRR (FOXG_00831), *F. verticillioides* TRR (FVEG_00627), *Ustilago maydis* TRR2 (UMAG_03763), *Aspergillus nidulans* TRR (CAL36645), *Magnaporthe oryzae* TRR (MGG_01284), *Schizosaccharomyces pombe* TRR (SPBC3F6.03), *Saccharomyces cerevisiae* TRR1/2 (NP_010640/NP_011974).


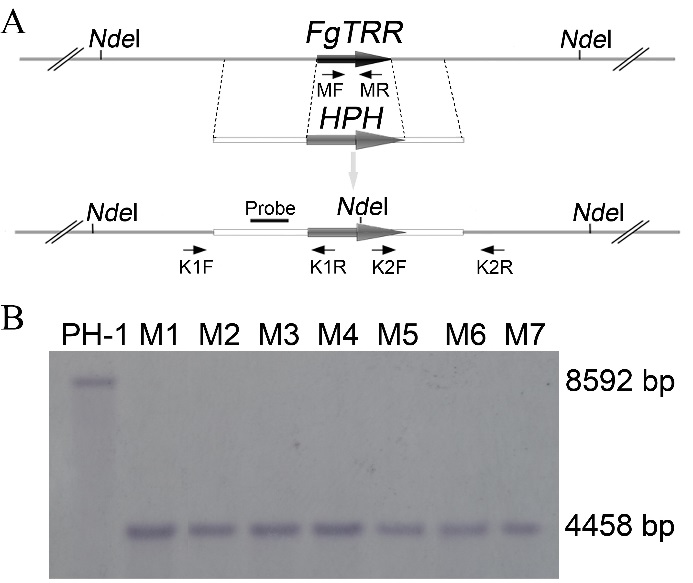

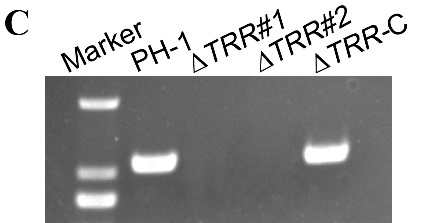


**FIGURE S2** **|** Schematic diagram and identification of *FgTRR* deletion mutants. **(A)** Deletion strategy used for *FgTRR*. The gene replacement cassette contains a hygromycin-phosphotransferase (HPH) gene. **(B)** Southern blot analysis of seven candidate *FgTRR* deletion transformants. Genomic DNA was digested with *Nde*I. **(C)** PCR analysis of *FgTRR* complementation strain.

**Table S1** **|** Primers used in this study

| Primer | Sequence (5′-3′) | | Application |
| --- | --- | --- | --- |
| TRR-A1  TRR-A2 | | AGCATCTGGCGACAGGGTA TTGACCTCCACTAGCTCCAGCCAAGCCTGGCGGTGTTGGAAGGAG | amplify *FgTRR* 5′ flank sequence |
| TRR-B1 | | GAATAGAGTAGATGCCGACCGCGGGTTTAGACTTGCCCCATTTTAGACAT | amplify *FgTRR* 3′ flank sequence |
| TRR-B2 | | CCAGCACATCCACGCCTA |  |
| HYG-F | | GGCTTGGCTGGAGCTAGTGGAGGTCAA | amplify *HPH* N-terminal sequence |
| HY-R | | AACCCGCGGTCGGCATCTACTCTATTC |  |
| YG-F | | GATGTAGGAGGGCGTGGATATGTCCT | amplify *HPH* C-terminal sequence |
| HYG-R | | GTATTGACCGATTCCTTGCGGTCCGAA |  |
| TRR-K1F | | AGCATCTGGCGACAGGGTA | for identification of *FgTRR* deletion transformants |
| TRR-K1R | | CAGCGGGCAGTTCGGTTT |  |
| TRR-K2F  TRR-K2R  TRR-MF  TRR-MR  tTRR-F  tTRR-R  EF1α-F  EF1α-R  GAPDH-F  GAPDH-R  TRI5-F  TRI5-R  TRI6-F  TRI6-R  TRI10-F  TRI10-R  TRR-CF  TRR-CR  GFP-F  GFP-R  AurF-F  AurF-R  AurO-F  AurO-R  AurJ-F  AurJ-R  PKS12-F  PKS12-R  gip1-F  gip1-R  gip2-F  gip2-R | | TCGTTATGTTTATCGGCACTTT  CACTACTACTCTTTCCGCTTCCTC TGGGATGTTACAGTTATCGGTTCT  TTAGTTGGGGTTGGGTCGC GCACAGACGGGATTGGAAAA TTGAAGGACGACGGACGATA  CAAGATTGGCGGTATTGGAAC  AGGAGGGTAGTCGGTGAAAGC  CTTACTGCCTCCACCAACTG  TGACGTTGGAAGGAGCGAAG  GAGTGTTTCATGCATGGCTACGTC  CTGAGCCTCCTTCACATCGTCC  CTGAGGGCATTCTGAGTAGCGACA  CGTTATGTTTATCGGCACTTTG  GCGACAGGAGCAAGAACATAA  GGCGGCGTAAATCTGAGTG  CTCACTATAGGGCGAATTGGGTACTCAAATTGGTTCCGAACAGGTAAGTTTACTCCATA CACCACCCCGGTGAACAGCTCCTCGCCCTTGCTCACATCCCGACACCAGCAAGG  CTCACTATAGGGCGAATTGGGTACTCAAATTGGTT CCGAACAGGTAAGTTTACTCCATA  CACCACCCCGGTGAACAGCTCCTCGCCCTTGCTCACGTTGGGGTTGGGTCGCTC  ATCTTCAGTCTTGACCATCCC  TACCCAAGATGTTCTGGCAA  AACAGCCTCAGGGCAATGA  TGACCAGACATCCGAGGTGAT  AAAAAGCAGCCAAGGAGCAT  TTCTGATGACACGCTCCCGTA  TGGTGTAGATGCTGTTCGTGT  TGAACTTTTCGAGGACGGAT  TGCGGTATCAGGTCACAAA  ATCAAAGTCTCCCACCGTGAA  CACCAGCCCTACACCATCTAA  TTTCCAAAGCGAGAAACAGC | for identification of *FgTRR* deletion transformants  for identification of *FgTRR* deletion transformants  amplify *FgTRR* probe sequence  for identification of cDNA  quantitative RT-PCR analysis  quantitative RT-PCR analysis  quantitative RT-PCR analysis  quantitative RT-PCR analysis  *FgTRR* complementation  for subcellular localization  quantitative RT-PCR analysis  quantitative RT-PCR analysis  quantitative RT-PCR analysis  quantitative RT-PCR analysis  quantitative RT-PCR analysis  quantitative RT-PCR analysis |
